# Local infiltration of tramadol as an effective strategy to reduce post-operative pain: a systematic review protocol and meta-analysis

**DOI:** 10.1186/s13643-020-01419-1

**Published:** 2020-07-13

**Authors:** Maria Beatrice Passavanti, Giacomo Piccinno, Aniello Alfieri, Sveva Di Franco, Pasquale Sansone, Giuseppe Mangoni, Vincenzo Pota, Caterina Aurilio, Maria Caterina Pace, Marco Fiore

**Affiliations:** Department of Women, Child and General and Specialized Surgery, University of Campania “Luigi Vanvitelli”, Piazza L. Miraglia 2, 80138 Naples, Italy

## Abstract

**Objective:**

The purpose of this review is to evaluate the use and effectiveness of the local administration of tramadol in reducing post-operative pain during surgical interventions.

**Methods:**

The PubMed, EMBASE and Cochrane Central Register of Controlled Trials (CENTRAL) databases will be searched for this review. This systematic review will include studies evaluating the clinical efficacy of the local infiltration of tramadol, with no study design restrictions. Only studies that present clear descriptions of local tramadol administration are published in peer-reviewed journals in the English, Italian, Spanish, French, Portuguese or German language and are published in full will be taken into consideration. A meta-analysis will be performed when there is sufficient clinical homogeneity among the retrieved studies, and only randomized controlled studies and quasi-randomized controlled studies will be included. The Grading of Recommendations Assessment, Development and Evaluation (GRADE) approach will be used to assess the certainty in the evidence. If a quantitative analysis cannot be conducted, a qualitative description of the results of the retrieved studies will be provided.

**Results:**

A high-quality synthesis of the current evidence on the local administration of tramadol for managing post-surgical pain will be illustrated using subjective reports and objective measures of performance. The primary outcomes will include the magnitude of post-operative pain intensity improvement, with improvement being as defined by a reduction by at least 2 points in the visual analogue scale (VAS) score or numerical rating scale (NRS) score. The secondary outcomes will be the magnitude of reduction in tramadol rescue doses and in other analgesic drug doses.

**Conclusion:**

This protocol will present evidence on the efficacy of tramadol in relieving post-surgical pain.

**Systemic review registration:**

PROSPERO CRD42018087381

## Background

Several surgical procedures are performed every day worldwide, and most of them are painful or uncomfortable for patients. Currently, due to advances in knowledge and anaesthetic techniques, many diagnostic procedures and an increasing number of surgical procedures can be performed under local anaesthesia. A ‘short’ procedure may be described as an intervention that is anticipated to take fewer than 60 min and can be performed safely in day surgery [1]. The prevention and management of post-operative pain are essential for the comfort of patients, and inadequate treatments can lead to major complications after surgery. Post-operative pain is defined by a non-zero score for the NRS (numerical rating scale), VAS (visual analogue scale) or other age-appropriate pain assessment tools at the end of the surgical procedure. Historically, pain has been arbitrarily considered either acute or chronic, and different mechanisms are believed to be involved in the development of every case of pain. Recent research, however, has indicated that we should view the two types of pain on a continuum with the same underlying process [2]. Fletcher et al*.* reported that every 10% increase in the time spent experiencing severe post-operative pain was associated with a 30% increase in chronic pain at 12 months after surgery. The use of multimodal perioperative and intraoperative techniques for pain control may decrease the incidence of acute pain and related complications such as chronic pain [3]. Local anaesthesia offers an indisputable advantage regarding intraoperative and post-operative pain control, with minimal systemic side effects and good patient tolerability [4]. Conventional local anaesthetics are made up of three common elements: an aromatic ring, an amino group and an ester or amide bond. When injected, local anaesthetics bind to the open form of the Na+ channel from the cytoplasmic side of the neuronal membrane, preventing the depolarization and the propagation of the signal. Local anaesthetics can be injected either at the surgical site (subcutaneous, submucosal, intra-articular) or at the nerves that innervate the surgical site region (troncular anaesthesia, plexus block, plane block). Although local anaesthetics are generally safe, these agents can be toxic if administered inappropriately, and in some cases, they may lead to unintended reactions, even when they are properly administered. Because of their chemical characteristics, local anaesthetics can produce various toxic effects in many tissues, especially the heart and brain [5]. In most reports, the rate of severe cases of toxicity (convulsive seizures with or without cardiac events) is approximately 1:10,000 for epidural analgesia and approximately 1:1000 for peripheral nerve blocks [6]. These toxic events result from the direct injection of such drugs into a vascular space or the absorption of these drugs by surrounding tissues. When these drugs are directly injected into a vascular space, symptoms occur within a few minutes. In contrast, when they are absorbed by surrounding tissues, symptoms may be delayed by many minutes or even hours [7]. The toxicity of local anaesthetics can be either local or systemic. Local adverse effects include neurovascular manifestations such as prolonged anaesthesia and paresthesia, which can be irreversible. Systemic adverse events include hypertension and tachycardia, conduction defects and arrhythmias, tinnitus, tonic-clonic seizures, decreased level of consciousness, apnoea and cardiac arrest.

Compared with local anaesthetics, tramadol seems to have fewer side effects and good efficacy and safety. In 1998, Pang and colleagues described for the first time the anaesthetic property of commercially available tramadol when it is injected intradermally [8]. In 2013, Yahya and Al-Haideri used it for the first time as an infiltrative anaesthetic for tooth extraction [9].

To date, according to studies on rats, we suppose that tramadol has an early local analgesic effect, decreasing mechanical hyperalgesia induced by plantar incisions. This analgesic effect is not mediated by peripheral opioid receptors.

Tramadol modifies both motor and sensory neural conduction with a mechanism similar to that of lidocaine and acts on the voltage-dependent sodium channel (NaV 1.2).

A high concentration of tramadol can indirectly and reversibly suppress sodium inward currents, blocking voltage-gated sodium channels (NaV 1.2) [10].

Some authors have reported that tramadol can interfere with nerve conduction, increasing the concentration of extracellular calcium (Ca+2) and raising the activation threshold of voltage-dependent channels [11].

Furthermore, it has been reported that the presence of a high Ca+2 concentration in the external medium increases tramadol activity.

Actually, the local anaesthetic effect of tramadol is weaker than that of lidocaine, but tramadol has fewer side effects and a lower risk of adverse events if it is accidentally injected inside a peripheral nerve [10].

There is evidence in the literature on the use of tramadol as a local anaesthetic, but to date, only the systemic administration of tramadol has been approved. In addition, limitations regarding paediatric use were recently announced: On September 21, 2015, the US Food and Drug Administration (FDA) issued a drug safety communication on the use of tramadol in children aged 17 years and younger because of the rare but serious risk of slowed or difficult breathing. This risk may be increased in children treated with tramadol for post-surgical pain due to tonsillectomy and/or adenoidectomy [12]. Subsequently, on April 20, 2017, the FDA issued a drug safety communication with a new contraindication for the use of tramadol in children younger than 18 years to treat post-surgical pain caused by tonsillectomy and/or adenoidectomy [13]. Unlike the FDA, the European Medicines Agency (EMA) does not contraindicate tramadol use in children but recommends extreme caution when it is administered to children for post-operative pain relief. Tramadol use should be accompanied by close monitoring for symptoms of opioid toxicity, including respiratory depression [14].

### Description of the intervention

Tramadol is an atypical synthetic opioid analgesic available on the market in a multitude of formulations. The analgesic potency of tramadol is approximately 10% of that of morphine and is considered a weak opioid because of its relatively low affinity for the μ-opioid receptor [15]. Compared with pure opioid agonists at equianalgesic doses, tramadol is significantly less likely to lead to respiratory depression and has a smaller effect on gastrointestinal motor function [16–23]. Additionally, when tramadol is administered by the parenteral route, the risk for adverse events is reduced [24]. Tramadol and its active metabolite bind to μ-opioid receptors in the central nervous system, resulting in the inhibition of ascending pain pathways and the reuptake of norepinephrine and serotonin, which are involved in the descending inhibitory pain pathway and associated with pain relief [25]. In addition, tramadol has been indicated in both clinical and laboratory studies to exert a local anaesthetic effect on peripheral nerves. The mechanism of action of tramadol seems to be similar to that of local anaesthetics, the blocking voltage-gated sodium channels [26].

## Methods

The review protocol was registered on PROSPERO (The International Prospective Register of Systematic Reviews), and the registration number is CRD42018087381 [27]. The last literature search will be carried out in June 2020, and the study is expected to be completed in November 2020. Ethical approval is unnecessary because we will extract published data.

### Selection criteria

#### Types of studies

This review will consider studies evaluating the clinical efficacy of the local infiltration of tramadol, with no study design restrictions. Only studies that clearly describe local tramadol administration, are published in peer-reviewed journals in the English, Italian, Spanish, French, Portuguese or German language and are published in full will be taken into consideration. Studies describing tramadol administration for non-surgical procedures will be excluded; reviews, abstracts, letters and comments will be excluded. A meta-analysis will be performed when there is sufficient clinical homogeneity among the retrieved studies, and only randomized controlled studies and quasi-randomized controlled studies will be included.

#### Types of participants

This review will consider all relevant studies in humans undergoing surgical interventions in which the use of tramadol as a local anaesthetic is clearly described. In vitro and animal studies will be excluded.

#### Types of interventions

This review will consider studies in which there is a clear description of tramadol use as local or locoregional anaesthetic. We will describe the efficacy of the local administration of this drug through subcutaneous, submucosal or intra-articular administration routes in controlling post-surgical pain. Using these administration routes, we will analyse the local action of tramadol in a circumscribed region, that is, the surgical incision site. Furthermore, studies concerning the use of tramadol as a locoregional anaesthetic (e.g. intrathecal administration or locoregional nerve blocks due to the direct action of tramadol on nervous plexus) alone or as an adjuvant to other anaesthetics will be considered because when the drug is administered by a locoregional technique, it may act directly on the central nervous system. We limited the types of interventions to increase the level of between-study homogeneity; therefore, studies regarding the oral administration, intravenous administration or intramuscular administration of tramadol will be excluded because the drug has systemic effects with these types of administration. Studies concerning the use of tramadol for non-surgical procedures will be excluded.

#### Types of comparator/control group(s)

The comparator will be the administration of local anaesthetics or a placebo with the same mode of administration as used for tramadol for post-surgical pain control. Studies in which different administration routes were used for tramadol and the comparator(s) will be excluded.

#### Types of outcome measures

The primary outcome will be the magnitude of post-operative pain control, with successful pain control being defined as a reduction by at least 2 points or by achieving a score of < 3 on the VAS, NRS or if necessary, another age-appropriate pain assessment tool.

The secondary outcomes will be the effects on drug consumption and vital signs.

Regarding drug consumption, the following factors will be considered:

1. The magnitude of reduction in tramadol rescue doses
2. The magnitude of reduction in other analgesic drug doses

Regarding vital signs, the following factors will be considered:

3. Tachycardia (heart rate > physiological range by age)
4. Hypertension (blood pressure > physiological range by age)
5. Desaturation (SpO2 < physiological range by age) with/without the occurrence of respiratory depression (requiring endotracheal intubation) and/or ICU admission.

### Data sources

The PubMed, EMBASE and Cochrane Central Register of Controlled Trials (CENTRAL) databases will be used for this review. The reference lists of all the identified reports and articles will be searched as well with backward and forward citation tracking. No restrictions on the dates of publication of the studies will be applied. The reference lists of all the included studies will be screened for additional eligible studies that were not identified by previous searches.

### Search strategy

The search strategy and the search string will be formulated following the PICO method.

In this review, a research strategy based on three steps has been designed to find and analyse only studies published in peer-reviewed journals.

The first search will be conducted in PubMed, followed by a detailed examination of the titles, abstracts and the index terms used to describe the articles. The information on the complete search strategy will be derived from previous research. The proposed search strategy is detailed in Table 1.

**Table 1:** PubMed search strategy (conducted on June 9, 2020)

Search	Query	Records retrieved
#1	((“anesthetics, local”[Pharmacological Action] OR “anesthetics, local”[MeSH Terms] OR (“anesthetics”[All Fields] AND “local”[All Fields]) OR “local anesthetics”[All Fields] OR (“local”[All Fields] AND “anesthetic”[All Fields]) OR “local anesthetic”[All Fields] OR “anesthesia, local”[MeSH Terms] OR (“anesthesia”[All Fields] AND “local”[All Fields]) OR “local anesthesia”[All Fields] OR (“local”[All Fields] AND “anesthetic”[All Fields])) AND (“tramadol”[MeSH Terms] OR “tramadol”[All Fields])) OR (((“anesthetics, local”[Pharmacological Action] OR “anesthetics, local”[MeSH Terms] OR (“anesthetics”[All Fields] AND “local”[All Fields]) OR “local anesthetics”[All Fields] OR (“local”[All Fields] AND “anesthetic”[All Fields]) OR “local anesthetic”[All Fields] OR “anesthesia, local”[MeSH Terms] OR (“anesthesia”[All Fields] AND “local”[All Fields]) OR “local anesthesia”[All Fields] OR (“local”[All Fields] AND “anesthetic”[All Fields])) AND (“pain, postoperative”[MeSH Terms] OR (“pain”[All Fields] AND “postoperative”[All Fields]) OR “postoperative pain”[All Fields] OR (“postoperative”[All Fields] AND “pain”[All Fields]))) AND (“tramadol”[MeSH Terms] OR “tramadol”[All Fields]))	556

A second search will be performed using the keywords and index terms identified through the following databases: PubMed, EMBASE and CENTRAL.

A third search will be performed using the bibliographies of the identified reports. The search will not include any language restrictions. Nevertheless, if the title/abstract of a study is in English and the full text is in another language, the study will be considered if the text is in the English, Italian, Spanish, French, Portuguese or German language.

### Data collection and analysis

#### Screening of the studies

Studies will be selected by a two-step process. Initially, the studies will be screened according to the titles and abstracts. Subsequently, the full text will be read for the final inclusion process. Duplicates will be excluded. Microsoft Excel software will be used to manage the study selection process and to remove all duplicates. Two authors will independently evaluate the eligibility of the studies. When there is missing information, the authors of the primary studies will be contacted. Explanations for the exclusion of full-text articles that do not meet the inclusion criteria will be recorded and reported in the review. Any disagreements that arise between the two authors during the study selection process will be resolved by discussion or with a third reviewer. Every result from each step of the search will be reported in full in the final report and presented in a Preferred Reporting Items for Systematic Reviews and Meta-analyses (PRISMA) flow diagram [28].

#### Data extraction

All identified citations will be imported into Endnote X8.0 (Clarivate Analytics, PA, USA), and duplicates will be removed. Data will be extracted from the included studies using a standardized data extraction tool by two independent reviewers; these data will include specific details about the populations, study methods, interventions and outcomes of significance to the review objective. Furthermore, patient characteristics, such as age, sex, ASA class and intervention characteristics, such as the dose, timing of administration, route of administration and pharmacological combination, will be extracted.

#### Quality of the studies

Two authors will independently evaluate the methodological quality of the retrieved studies before inclusion in the review. Study quality will be independently assessed by two authors. The Newcastle-Ottawa quality assessment form will be used to assess the quality of the observational studies, while the Cochrane tool (Rob2) will be used to assess the quality of the randomized controlled trials (RCTs) [29]. Any disagreements between the reviewers will be resolved by discussion with a third reviewer**.**

If required, the authors of the studies will be contacted to request missing or additional data for explanation. Any disagreements between the authors will be resolved by discussion or with a third author. The results will be reported in narrative form and summarized in tables.

#### Management of missing data

If there are missing or incomplete data for the primary results, we will contact the corresponding authors for the missing data. If the missing data cannot be obtained, it will be included in a narrative analysis.

### Data synthesis

#### Narrative analysis

We may conduct a narrative synthesis if a meta-analysis is not appropriate.

#### Meta-analysis

Risk differences (RDs) will be used as the meta-analytic measure of the association between the administration of tramadol and post-surgical pain relief. We will calculate risk differences and corresponding 95% confidence intervals (CIs) using a 2 × 2 table.

#### Analysis of subgroups or subsets

If possible, a subgroup analysis will be conducted for the following factors:

1. The route of administration used (local: (a) subcutaneous, (b) submucosal, (c) intra-articular; locoregional: (d) intrathecal, (e) nerve block)
2. The age of the patients treated (e.g. children, adults, elderly people)

#### Assessment of heterogeneity

Heterogeneity between studies will be assessed by using the *Q* and *I*^2^ statistics, and the *I*^2^ statistic is defined as the proportion of total variance observed among the studies that is attributed to differences among the studies rather than to sampling error. *I*^2^ values of 25%, 50% and 75% correspond to cut-off points for low, moderate and high degrees of heterogeneity, and a *P* value of less than 0.10 for the *Q* statistic will be considered significant. If the overall heterogeneity is significant, a random-effects model will be used; otherwise, a fixed-effects model will be used.

#### Assessment of the quality of evidence

The Grading of Recommendations, Assessment, Development and Evaluation (GRADE) approach for grading the certainty of evidence will be used to assess the certainty of the findings, and a summary of findings (SoF) will be created using GRADE-Pro GDT software. The SoF will present the following information where appropriate: absolute risks for the treatment and control, estimate of the relative risk and ranking of the quality of the evidence based on the risk of bias, directness, heterogeneity, precision and risk of publication bias of the review results. All primary outcomes will be included in the summary of findings.

## Discussion

The purpose of this protocol is to investigate the potential off-label use of a drug commonly used in clinical practice for systemic administration. Its use as an adjuvant in local anaesthesia is described in the literature [30–32] and may be useful in adequately managing post-surgical pain, thereby reducing the intake of drugs that may have immediate and delayed adverse events. The activation of nociceptors is caused by different types of lesions and is related to the release of numerous inflammatory mediators that are not yet fully known [33], such as the local analgesic effect of tramadol. Clinical studies should find a solution to mitigate analgesic use [34], and this study contributes to this field of research. As there are concerns about tramadol use in children, due to the potential risk of respiratory depression, another issue that this systematic review will evaluate is the safety of the drug.

## Data Availability

The data is available upon request from the corresponding author.

## Abbreviations

CENTRAL: The Cochrane Central Register of Controlled Trials
CI: Confidence interval
GRADE: Grading of Recommendations, Assessment, Development and Evaluation
NRS: Numerical rating scale
PRISMA: Preferred Reporting Items for Systematic Reviews and Meta-analyses
PROSPERO: The International Prospective Register of Systematic Reviews
RCTs: Randomized controlled trials
RDs: Risk differences
SoF: Summary of findings
VAS: Visual analogue scale
